# Characterizing and Improving pET Vectors for Cell-free Expression

**DOI:** 10.3389/fbioe.2022.895069

**Published:** 2022-06-23

**Authors:** Kara Jew, Philip E. J. Smith, Byungcheol So, Jillian Kasman, Javin P. Oza, Michael W. Black

**Affiliations:** ^1^ Biological Sciences Department, California Polytechnic State University, San Luis Obispo, CA, United States; ^2^ Chemistry & Biochemistry Department, California Polytechnic State University, San Luis Obispo, CA, United States

**Keywords:** cell-free, protein synthesis, pET30, template, *in vitro*, translation

## Abstract

Cell-free protein synthesis (CFPS) is an *in vitro* process that enables diverse applications in research, biomanufacturing, point-of-care diagnostics, therapeutics, and education using minimal laboratory equipment and reagents. One of the major limitations of CFPS implementation is its sensitivity to plasmid type. Specifically, plasmid templates based on commonly used vector backbones such as the pET series of bacterial expression vectors result in the inferior production of proteins. To overcome this limitation, we have evaluated the effect of expression cassette elements present in the pET30 vector on protein production across three different CFPS systems: NEBExpress, PURExpress, and CFAI-based *E. coli* extracts. Through the systematic elimination of genetic elements within the pET30 vector, we have identified elements that are responsible for the poor performance of pET30 vectors in the various CFPS systems. As a result, we demonstrate that through the removal of the *lac* operator (*lacO)* and N-terminal tags included in the vector backbone sequence, a pET vector can support high titers of protein expression when using extract-based CFPS systems. This work provides two key advances for the research community: 1) identification of vector sequence elements that affect robust production of proteins; 2) evaluation of expression across three unique CFPS systems including CFAI extracts, NEBexpress, and PURExpress. We anticipate that this work will improve access to CFPS by enabling researchers to choose the correct expression backbone within the context of their preferred expression system.

## Introduction

Cell-free protein synthesis (CFPS) provides an on-demand protein expression platform that is compatible with circular plasmids as well as linear DNA and RNA templates (Jewett and Swartz, 2004; Gregorio et al., 2019; Asahara et al., 2021; McSweeney and Styczynski, 2021; Batista et al., 2022). The use of CFPS bypasses the need to maintain living cells, therefore, all cellular energy and machinery can be directed toward protein synthesis. The open nature of the cell-free platform allows users greater control of the reaction conditions than *in vivo* expression platforms. CFPS also enables the expression of cytotoxic and complex proteins that may otherwise be difficult to express in living cells (Jewett and Swartz, 2004; Pardee et al., 2016; Dopp et al., 2019; Garenne et al., 2019; Jin et al., 2019; Kay and Jewett, 2020). Recently, improvements in the upstream and downstream processing of cell lysates from the widely adopted *E. coli* platform have led to more consistent results and an increased shelf life of the reaction mixtures (Smith et al., 2014; Kwon and Jewett, 2015; Cole et al., 2020; Gregorio et al., 2020; Levine et al., 2020). Due to these benefits, CFPS systems are enabling a variety of academic research efforts, biotechnology innovations, and large scale biomanufacturing (Pardee et al., 2016; Huang et al., 2018; Khambhati et al., 2019; Kightlinger et al., 2019; Choi et al., 2020; Liu et al., 2020; Silverman et al., 2020; Williams et al., 2020; Burrington et al., 2021a; Brookwell et al., 2021; Si et al., 2021).

Barriers to access have reduced significantly as CFPS systems have become commercially available in the form of kits derived from lysates of a variety of chassis organisms, as well as reconstituted systems (Shimizu et al., 2005). For this study, the New England Biolab’s NEBExpress and NEB PURExpress kits were used alongside our in-house *E. coli* lysate-based CFAI system to assess the effects that distinct vector elements have on protein synthesis (Levine et al., 2020; Smith et al., 2021; Mullin et al., 2022). Both the NEBExpress and CFAI systems utilize crude *E. coli* extracts. In contrast, the NEB PURExpress system is reconstituted with purified components of the *E. coli* translation machinery. The purified systems are an important part of the CFPS biotechnology portfolio since they provide protein expression conditions in which protease and nuclease activity is minimized (Shimizu et al., 2001) to preserve nucleic acid templates and protein products.

The CFPS community has systematically reduced many of the bottlenecks that limited the broad utility of CFPS ushering the biotechnology’s renaissance over the last 20 years. However, compatibility of DNA templates in the CFPS system continues to remain a limit for the robust production of target proteins (Romantseva and Strychalski, 2020). In the *E. coli*–based CFPS platform, commonly used pET series expression vectors have been observed to result in significantly lower protein titers and yields when compared to the alternate vectors such as pJL1 (Zhang et al., 2018; Colant et al., 2021). The pJL1 vector (Addgene #69496 and #102634) is derived from the pY71 vector, which was a simplified version of the pK7 plasmid. This plasmid lineage has been successfully utilized for CFPS and has set the benchmarks for CFPS applications for over a decade (Swartz et al., 2004; Bundy and Swartz, 2010). The importance of expression vectors has also been demonstrated in Streptomyces-based cell-free systems (Xu et al., 2022). *In vivo* studies in *E. coli* have identified features within the pET series of expression vectors that hinder protein expression yields (Shilling et al., 2020). The *in vivo* study determined that an incomplete T7 promoter found in pET28a decreased sfGFP production. This truncated T7 promoter was also identified in 88 of the 103 pET expression vectors. Such efforts are needed for *in vitro* expression given the precedence for variation in sequence elements found in expression vectors being consequential for expression yields. We first established that the vector used in this study, pET30, contains the complete T7 promoter. The goal of this study was to examine additional features of the pET expression vector series that may have an impact on protein yields in CFPS systems. We assessed the effects of the pET30 *lacO* and the N-terminal tags (6x poly-histidine tag and S tag) on sfGFP expression. Four versions of the pET30 vector were constructed with and without *lacO* and N-terminal His-tag. The expression of sfGFP across three CFPS expression systems was then determined through fluorescence evaluate the impact of these sequence elements.

## Materials and Methods

### Strains and Growth Conditions

*E. coli* strains BL21(DE3) and MC1061 were used in this study. Cultures were aerobically grown at 37°C in Luria Bertaini (LB) broth or plates. Kanamycin (30 μg/ml) was added to the media for cultures containing pET30-derived vectors and pJL1-sfGFP. The BL21(DE3) strain was used to prepare CFAI-based CFPS extracts as previously described (Levine et al., 2020; Smith et al., 2021; Mullin et al., 2022). The CFAI media auto-induces T7 RNAP expression during cell growth, and cells are harvested at high ODs. The MC1061 strain was used as the host for cloning variations of the pET30 expression plasmids. All transformations were performed via electroporation with 40 μl of electrocompetent cells and approximately 30 ng of DNA using the BTX Electro Cell Manipulator 600 (Harvard Apparatus Inc.; 2.45 kV, 129 Ω). Immediately after electroporation, cells were incubated with 500 μl SOC recovery medium for 1 h at 37°C, plated on LB-kanamycin plates, and incubated at 37°C for 18–24 h.

### Molecular Techniques

The polymerase chain reactions (PCR) were performed in 20 µl volumes with Phusion Flash High-Fidelity PCR 2X Master Mix (Thermo Scientific, Rockford, IL, United States) containing 0.2 ng of template DNA and a final primer concentration of 0.1 µM. The vector and inserts used to construct the pET30 variations were amplified with forward and reverse primers noted in Table 1. The thermocycling parameters included a 1-min denaturation at 98°C followed by 30 cycles of 10 s at 98°C, 30 s at various annealing temperatures, and 15 s per kb of expected product at 72°C. The reaction ended with a final 5-min extension step at 72°C and hold at 4°C.

**TABLE 1:** Primers used to construct pET30 expression vectors. Primers for amplification of insert and vector backbones used in Gibson assembly to construct pET30-T7-sfGFP, pET30-lacO-sfGFP, pET30-His-sfGFP, and pET30-lacO-His-sfGFP.

Primer sequences		T_m_	Annealing
pET30-T7-sfGFP			
Insert: T7-Pro-Gib-F	ccg​cga​aat​taa​tac​gac​tca​cta​tag​g	59°C	63°C
Insert: T7-Term-Gib-R	ctt​tca​gca​aaa​aac​ccc​tca​ag	56°C	
Vector: T7-Term-Gib-F	ctt​gag​ggg​ttt​ttt​gct​gaa​ag	56°C	63°C
Vector: T7-Pro-Gib-R	cct​ata​gtg​agt​cgt​att​aat​ttc​gcg​g	59°C	
pET30-T7-lacO-sfGFP			
Insert: RBS-sfGFP-F	ctt​taa​gaa​gga​gat​ata​cat​atg​agc​aaa​ggt​gaa​gaa​ctg	62°C	55°C
Insert: T7-Term-Gib-R	ctt​tca​gca​aaa​aac​ccc​tca​ag	56°C	
Vector: T7-Term-Gib-F	ctt​gag​ggg​ttt​ttt​gct​gaa​ag	56°C	63°C
Vector: pET-RBS-long-R	cat​atg​tat​atc​tcc​ttc​tta​aag​tta​aac​aaa​att​att​tct​aga​gg	58°C	
pET30-T7-His/S-sfGFP			
Insert: pET-RBS-F	gtt​taa​ctt​taa​gaa​gga​gat​ata​cat​atg	52°C	58°C
Insert: T7-Term-Gib-R	ctt​tca​gca​aaa​aac​ccc​tca​ag	56°C	
Vector: T7-Term-Gib-F	ctt​gag​ggg​ttt​ttt​gct​gaa​ag	56°C	58°C
Vector: pET-RBS-long-R	cat​atg​tat​atc​tcc​ttc​tta​aag​tta​aac​aaa​att​att​tct​aga​gg	58°C	
pET30-T7-lacO-His/S-sfGFP			
Insert: N-tag-sfGFP-F	cac​atg​gac​agc​cca​gat​ctc​atg​agc​aaa​ggt​gaa​gaa​ctg	69°C	63°C
Insert: T7-Term-Gib-R	ctt​tca​gca​aaa​aac​ccc​tca​ag	56°C	
Vector: T7-Term-Gib-F	ctt​gag​ggg​ttt​ttt​gct​gaa​ag	56°C	61°C
Vector: pET-No-Cut-DIC-R	aga​tct​ggg​ctg​tcc​atg​tg	58°C	

Gibson assembly was performed using 17 fmoles each of the amplified insert and vector fragments in a 6 µl reaction containing Taq ligase (4 U/µl), T5 exonuclease (0.02 U/µl), and Phusion DNA polymerase (0.025 U/µl) purchased from New England Biolabs (Ipswich, MA, United States). Each reaction was incubated 15 min at 50°C in 1X Gibson buffer (125 mM Tris-HCl pH 7.5, 6.25% PEG-8000 (w/v), 12.5 mM MgCl_2,_ 12.5 mM DTT, 2.5 mM dNTPs, and 1.25 mM NAD).

Cell-free protein synthesis reactions using in-house CFAI-based cell extracts were performed as described (Levine et al., 2020). Reactions using the PURExpress^®^ and NEBExpress^®^ kits (New England Biotech, Ipswich, MA, United States) were performed according to manufacturer’s instructions. All CFPS reactions were run in triplicate using sfGFP as the reporter protein.

### Quantification of Reporter Protein sfGFP

Fluorescence intensities of sfGFP from each CFPS reaction were measured in triplicate. Each measurement consisted of a solution of 48 µl of 0.05 M HEPES at pH 7.2 and 2 µl of the sfGFP CFPS reaction solution in a black 96 well plate. Each 50 µl solution’s fluorescence was then measured at an excitation wavelength of 485 nm and an emission wavelength of 528 nm. The quantity of sfGFP was then calculated using a previously developed standard curve (Levine et al., 2019).

## Results

**FIGURE 1:**
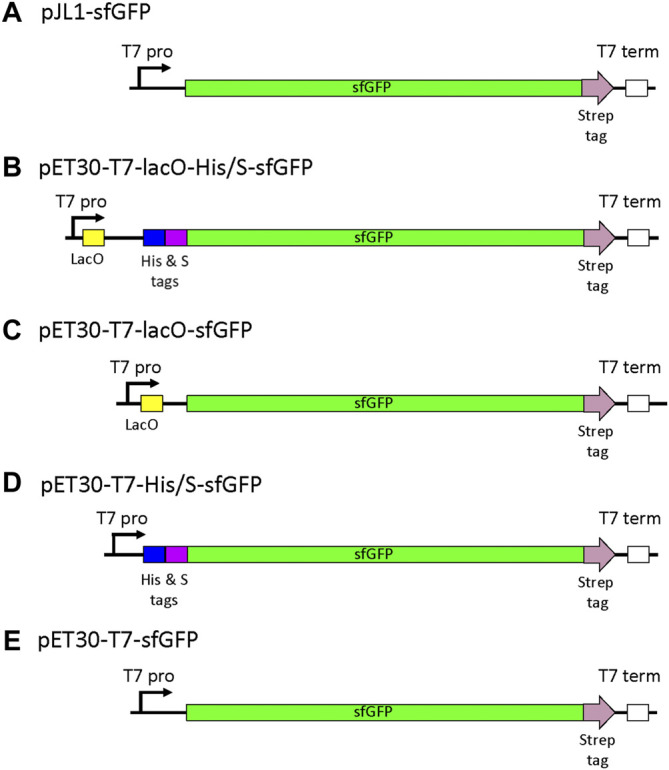
Schematic of the expression vectors studied. **(A)** pJL1-sfGFP represents the reference vector, **(B)** the pET30-T7-lacO-His/S-sfGFP cassette contains the T7 promoter (T7 pro) followed by *lacO* and a N-terminal purification tags (His and S tags) included in the pET30 vector backbone. Modifications of the pET30 expression cassette included the deletion of **(C)** the N-terminal His/S-tags, **(D)** *lacO,* and **(E)** both *lacO* and the N-terminal tags to assess the effects on sfGFP expression. Similar to pET30-T7-sfGFP, the pJL1-T7-sfGFP cassette includes the same T7 promoter but not the *lacO* nor the N-terminal His-tag. All templates used in this study were in their circular plasmid forms. While the backbone sequences are not displayed here, pET30a includes the *lacI* gene that is not present in the pJL1 backbone.

To systematically determine the effect of each constituent of the pET vector on CFPS, four vectors were constructed: pET30-T7-lacO-His/S-sfGFP (Addgene #180754), pET30-T7-lacO-sfGFP (Addgene #180756), pET30-T7-His/S-sfGFP (Addgene #180755), and pET30-T7-sfGFP (Addgene #180757). Graphic representations of the expression cassettes in each of the four plasmids are shown in Figure 1. As seen in Figure 1B, the gene encoding *sfGFP* is located downstream from both *lacO* and the N-terminal tags, and transcription is controlled by the T7 promoter. The effect on cell-free expression was measured as a function of removing the pET30 encoded N-terminal His & S tags (pET30-lacO-sfGFP), the *lacO* (pET30-His/S-sfGFP), or both N-terminal tags and the *lacO* sequences (pET30-T7-sfGFP). For this assessment, sfGFP expression from these plasmids were compared to the preferred pJL1-sfGFP reporter plasmid, which is also driven by the T7 promoter but does not include *lacO* or N-terminal His & S tags in the expression cassette. An alignment of the most relevant sequences that differ between the plasmids is provided in Supplementary Figure S1.

**FIGURE 2:**
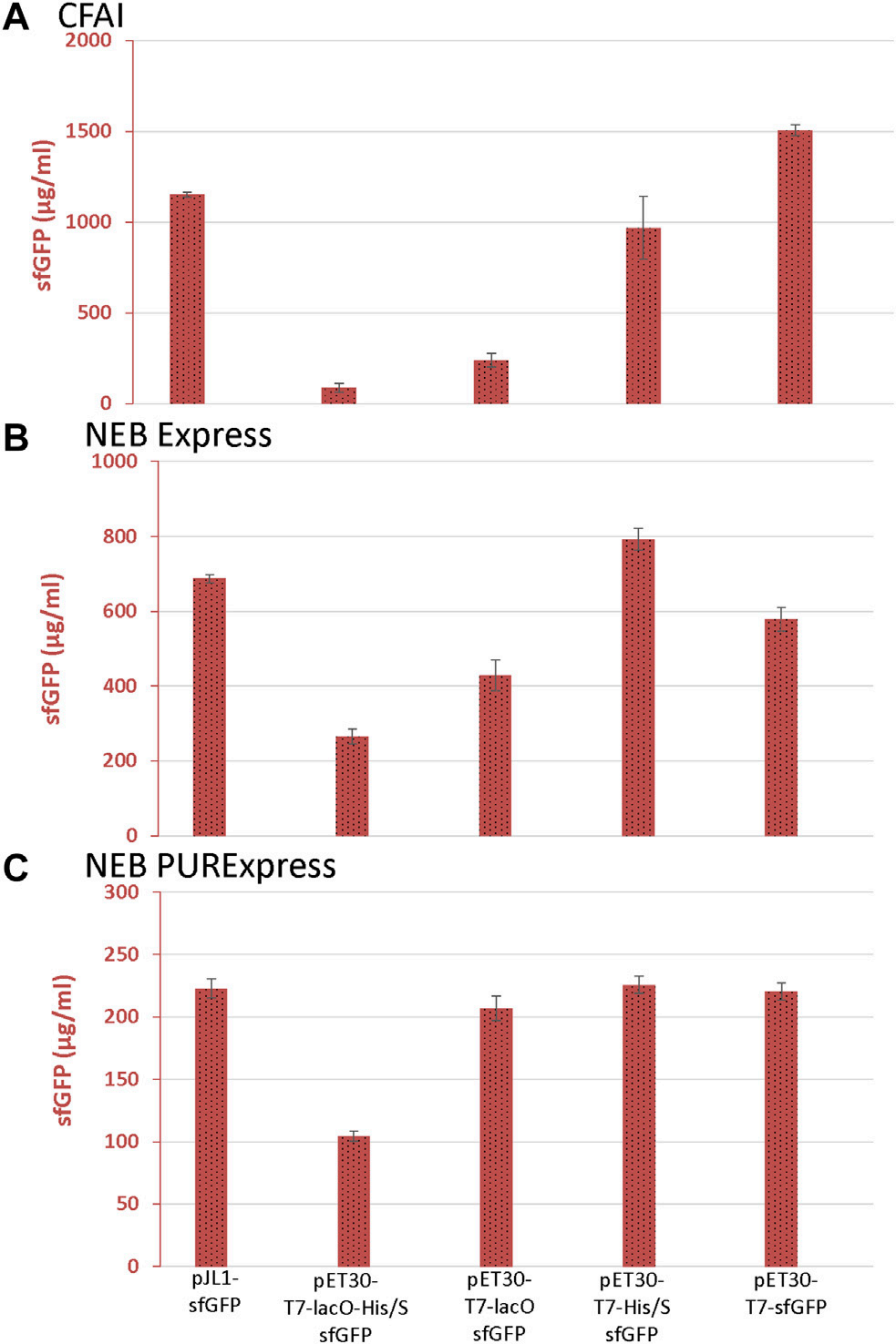
sfGFP expression from the CFAI-based, NEB Express, and NEB PURExpress CFPS systems. The sfGFP yields from **(A)** CFAI-based, **(B)** NEBExpress, and **(C)** NEB PURExpress CFPS systems determined by fluorescence. Data are presented as mean ± s.d. (*n* = 3).

In the CFAI-based CFPS system, removing *lacO* (pET30-T7-His/S-sfGFP), the N-terminal tags (pET30-T7-lacO-sfGFP), or both *lacO* and the N-terminal tags (pET30-T7-sfGFP) resulted in increased sfGFP expression (Figure 2A). The removal of *lacO* alone (pET30-T7-His/S-sfGFP) had a greater effect on improving sfGFP expression compared to the removal of the N-terminal tags alone (pET30-T7-lacO-sfGFP). For the NEBExpress system, there was an increase in sfGFP expression when la*cO* (pET30-T7-His/S-sfGFP), the N-terminal tags (pET30-T7-lacO-sfGFP), or the combination of both *lacO* and the N-terminal tags (pET30-T7-sfGFP) were removed from the pET30 vector (Figure 2B). Similar to the CFAI-based CFPS system, the removal of *lacO* alone (pET30-T7-His/S-sfGFP) had a greater impact on improving sfGFP expression compared to the removal of N-terminal tags alone (pET30-T7-lacO-sfGFP).

As observed with extract-based CFPS systems, sfGFP expression could also be improved for the NEB PURExpress system upon removing elements upstream of the reporter gene. The PURExpress system was less sensitive to the *lacO* element, lacking the repressor is a likely advantage of the purified system*.* sfGFP expression increased notably upon removal of the N-terminal tags (pET30-T7-lacO-sfGFP) and when both *lacO* and the N-terminal tags were removed (pET30-T7-sfGFP) (Figure 2C).

Overall, removing both *lacO* and the N-terminal tags enhanced the expression of sfGFP; however, the individual effects of *lacO* and the N-terminal tags differed between the three CFPS systems. In general, the removal of *lacO* alone had a more substantial impact on sfGFP fluorescence and expression in the extract-based CFAI and NEB Express systems whereas, in the NEB PURExpress CFPS system, the removal of the N-terminal tags had the greatest effect. Consistent with prior observations, fluorescence data also revealed much higher maximum yields of sfGFP through the CFAI-based CFPS system (>1,000 μg/ml) than in the NEB Express (∼800 μg/ml) and NEB PURExpress (∼200 μg/ml) CFPS systems (Burrington et al., 2021b).

## Discussion

The choice of expression vectors plays a critical role in CFPS as vectors may contain elements that negatively impact protein expression. When implementing pET vectors in CFPS, the removal of *lacO* and the N-terminal tags resulted in increased sfGFP production across all three expression systems tested. The removal of *lacO* appears to play a more significant role in sfGFP expression in the lysate-based CFAI and the NEB Express CFPS systems. This is likely due to the presence of lactose operon repressor present in the *E. coli* cell extracts and absent in the reconstituted PURExpress system. As the pET30 plasmids include a *lacI* expression cassette, residual *E. coli* RNA polymerase in the extracts may also lead to expression of supplemental LacI repressor directly from the vector. When *lacO* was present in the pET30 expression vector (pET30-T7-lacO-sfGFP and pET30-T7-lacO-His/S-sfGFP), there was a larger decrease in sfGFP yield in the CFAI system (Figure 2A) compared to the decrease in the NEB Express CFPS system (Figure 2B). These differences may be due to the nuanced methods in extract preparation between the two systems. According to NEB, the NEBExpress CFPS system is supplied with an optimized quantity of wild-type T7 RNA polymerase, which may produce mRNA transcripts more efficiently in the presence of the *lac* repressor. The NEBExpress system is generated from an *E. coli* strain which has a single copy of the lacI gene, which presumably expresses low levels of the Lac repressor protein. Optimized quantities of DNA template are therefore expected to exceed the number of Lac repressor molecules. Increasing DNA template quantities may be one possible solution to the Lac-based repression. The benefits observed from the removal of the *lacO* sequence may also be due to additional, more nuanced factors such as mRNA structure and stability, and possible impacts on translation initiation. While these and other factors are worth further study, the results of this work provide practical and actionable set of insights for researchers to either sub-clone a gene of interest into a CFPS-compatible vector, or selectively eliminate regions that may adversely impact CFPS expression from their preferred vector.

Purification is a typical goal of recombinant protein expression, so purification tags are often integrated into frequently used expression vectors. The presence of N-terminal His and S tags suppressed protein expression in all three CFPS systems to varying extents. Investigating the interplay between N-terminal tags and ribosome binding site (RBS) sequences may also be warranted (Salis et al., 2009; Zhang et al., 2021). To evaluate the importance of the RBS, we utilized the extremely useful tool De Novo DNA (www.denovodna.com) (Espah Borujeni et al., 2014; Espah Borujeni and Salis, 2016; Espah Borujeni et al., 2017). Notably, the RBS calculations do not correlate with the sfGFP expressions observed in our vectors, highlighting the importance of other molecular mechanisms must be at play for optimal expression in CFPS. Another consideration is that the presence of the His-tag may deplete the pool of L-histidine in the CFPS reactions, which could be further studied by either supplementing L-histidine to the reaction, or evaluating additional constructs in which the His-tag is moved to the C-terminus rather than removed. Given the importance of purification tags and the need to utilize them at the N-terminus, it will be important to further examine the effects of additional affinity tags. By investigating such effects, there may be an ideal purification tag that can be used to provide optimal protein expression in CFPS systems within the user’s expression vector of choice. When possible, a C-terminal tag may be preferred, but due to the context dependencies of biomolecular systems, these data provide evidence that users should evaluate a variety of construct designs that vary in the type and location of purification tags to achieve optimal protein expression. This work demonstrates that vector elements have substantial effects on CFPS yields. Furthermore, the effects of a given element are dependent on the context of the CFPS system in which the vector will be utilized. Our results nullify the hypothesis that pET vectors result in inferior protein expression due to their significantly larger size and provides further support for the role of specific elements that interfere with expression. Based on our findings, we are optimistic that researchers utilizing CFPS for protein expression will achieve improved yields by pairing an optimized vector with their preferred expression system.

## Data Availability

The original contributions presented in the study are included in the article/Supplementary Material, further inquiries can be directed to the corresponding authors.

## References

[B1] Asahara H. Magnelli P. Shi X. Tuckey C. Zhou Y. Samuelson J. C. (2021). “Guidelines for Nucleic Acid Template Design for Optimal Cell-free Protein Synthesis Using an Escherichia coli Reconstituted System or a Lysate-Based System,” in *Methods In Enzymology* Recombinant Protein Expression: Prokaryotic Hosts and Cell-free Systems. Editors Kelman Z. O’Dell W. B. (Academic Press), 351–369. 10.1016/bs.mie.2021.07.005 34752294

[B2] Batista A. C. Levrier A. Soudier P. Voyvodic P. L. Achmedov T. Reif-Trauttmansdorff T. (2022). Differentially Optimized Cell-free Buffer Enables Robust Expression from Unprotected Linear DNA in Exonuclease-Deficient Extracts. ACS Synth. Biol. 11, 732–746. 10.1021/acssynbio.1c00448 35034449

[B3] Brookwell A. Oza J. P. Caschera F. (2021). Biotechnology Applications of Cell-free Expression Systems. Life 11, 1367. 10.3390/life11121367 34947898 PMC8705439

[B4] Bundy B. C. Swartz J. R. (2010). Site-Specific Incorporation of P-Propargyloxyphenylalanine in a Cell-free Environment for Direct Protein−Protein Click Conjugation. Bioconjugate Chem. 21, 255–263. 10.1021/bc9002844 20099875

[B5] Burrington L. R. Baryal E. Hui K. Lambert E. Harding S. T. Oza J. P. (2021a). The Fold-Illuminator: A Low-Cost, Portable, and Disposable Incubator-Illuminator Device. Synthetic Syst. Biotechnol. 6, 95–101. 10.1016/j.synbio.2021.04.003 PMC8099501 33997359

[B6] Burrington L. R. Watts K. R. Oza J. P. (2021b). Characterizing and Improving Reaction Times for E. Coli-Based Cell-free Protein Synthesis. ACS Synth. Biol. 10, 1821–1829. 10.1021/acssynbio.1c00195 34269580

[B7] Choi S. Y. Rhie M. N. Kim H. T. Joo J. C. Cho I. J. Son J. (2020). Metabolic Engineering for the Synthesis of Polyesters: A 100-year Journey from Polyhydroxyalkanoates to Non-natural Microbial Polyesters. Metab. Eng. 58, 47–81. 10.1016/j.ymben.2019.05.009 31145993

[B8] Colant N. Melinek B. Teneb J. Goldrick S. Rosenberg W. Frank S. (2021). A Rational Approach to Improving Titer in Escherichia coli ‐based Cell‐free Protein Synthesis Reactions. Biotechnol. Prog. 37, e3062. 10.1002/btpr.3062 32761750

[B9] Cole S. D. Miklos A. E. Chiao A. C. Sun Z. Z. Lux M. W. (2020). Methodologies for Preparation of Prokaryotic Extracts for Cell-free Expression Systems. Synthetic Syst. Biotechnol. 5, 252–267. 10.1016/j.synbio.2020.07.006 PMC7398980 32775710

[B10] Dopp B. J. L. Tamiev D. D. Reuel N. F. (2019). Cell-free Supplement Mixtures: Elucidating the History and Biochemical Utility of Additives Used to Support *In Vitro* Protein Synthesis in E. coli Extract. Biotechnol. Adv. 37, 246–258. 10.1016/j.biotechadv.2018.12.006 30572024

[B11] Espah Borujeni A. Channarasappa A. S. Salis H. M. (2014). Translation Rate Is Controlled by Coupled Trade-Offs between Site Accessibility, Selective RNA Unfolding and Sliding at Upstream Standby Sites. Nucleic Acids Res. 42, 2646–2659. 10.1093/nar/gkt1139 24234441 PMC3936740

[B12] Espah Borujeni A. Salis H. M. (2016). Translation Initiation Is Controlled by RNA Folding Kinetics via a Ribosome Drafting Mechanism. J. Am. Chem. Soc. 138, 7016–7023. 10.1021/jacs.6b01453 27199273

[B13] Espah Borujeni A. Cetnar D. Farasat I. Smith A. Lundgren N. Salis H. M. (2017). Precise Quantification of Translation Inhibition by mRNA Structures that Overlap with the Ribosomal Footprint in N-Terminal Coding Sequences. Nucleic Acids Res. 45, 5437–5448. 10.1093/nar/gkx061 28158713 PMC5435973

[B14] Garenne D. Beisel C. L. Noireaux V. (2019). Characterization of the All‐ E. coli Transcription‐translation System myTXTL by Mass Spectrometry. Rapid Commun. Mass Spectrom. 33, 1036–1048. 10.1002/rcm.8438 30900355

[B15] Gregorio N. E. Kao W. Y. Williams L. C. Hight C. M. Patel P. Watts K. R. (2020). Unlocking Applications of Cell-free Biotechnology through Enhanced Shelf Life and Productivity of E. coli Extracts. ACS Synth. Biol. 9, 766–778. 10.1021/acssynbio.9b00433 32083847

[B16] Gregorio N. E. Levine M. Z. Oza J. P. (2019). A User's Guide to Cell-free Protein Synthesis. MPs 2, 24. 10.3390/mps2010024 PMC6481089 31164605

[B17] Huang A. Nguyen P. Q. Stark J. C. Takahashi M. K. Donghia N. Ferrante T. (2018). BioBits Explorer: A Modular Synthetic Biology Education Kit. Sci. Adv. 4. 10.1126/sciadv.aat5105 PMC6070312 30083608

[B18] Jewett M. C. Swartz J. R. (2004). Mimicking theEscherichia Coli Cytoplasmic Environment Activates Long-Lived and Efficient Cell-free Protein Synthesis. Biotechnol. Bioeng. 86, 19–26. 10.1002/bit.20026 15007837

[B19] Jin X. Kightlinger W. Hong S. H. (2019). Optimizing Cell-free Protein Synthesis for Increased Yield and Activity of Colicins. MPs 2, 28. 10.3390/mps2020028 PMC6632115 36358105

[B20] Kay J. E. Jewett M. C. (2020). A Cell-free System for Production of 2,3-butanediol Is Robust to Growth-Toxic Compounds. Metab. Eng. Commun. 10, e00114. 10.1016/j.mec.2019.e00114 31934547 PMC6951449

[B21] Khambhati K. Bhattacharjee G. Gohil N. Braddick D. Kulkarni V. Singh V. (2019). Exploring the Potential of Cell-free Protein Synthesis for Extending the Abilities of Biological Systems. Front. Bioeng. Biotechnol. 7, 248. Available at: https://www.frontiersin.org/article/10.3389/fbioe.2019.00248 (Accessed March 4, 2022). 10.3389/fbioe.2019.00248 31681738 PMC6797904

[B22] Kightlinger W. Duncker K. E. Ramesh A. Thames A. H. Natarajan A. Stark J. C. (2019). A Cell-free Biosynthesis Platform for Modular Construction of Protein Glycosylation Pathways. Nat. Commun. 10, 5404. 10.1038/s41467-019-12024-9 31776339 PMC6881289

[B23] Kwon Y.-C. Jewett M. C. (2015). High-throughput Preparation Methods of Crude Extract for Robust Cell-free Protein Synthesis. Sci. Rep. 5, 8663. 10.1038/srep08663 25727242 PMC4345344

[B24] Levine M. Z. Gregorio N. E. Jewett M. C. Watts K. R. Oza J. P. (2019). *Escherichia Coli*-Based Cell-Free Protein Synthesis: Protocols for a Robust, Flexible, and Accessible Platform Technology. J. Vis. Exp. (144), e58882. 10.3791/58882 30855561

[B25] Levine M. Z. So B. Mullin A. C. Fanter R. Dillard K. Watts K. R. (2020). Activation of Energy Metabolism through Growth Media Reformulation Enables a 24-Hour Workflow for Cell-free Expression. ACS Synth. Biol. 9, 2765–2774. 10.1021/acssynbio.0c00283 32835484

[B26] Liu R. Zhang Y. Zhai G. Fu S. Xia Y. Hu B. (2020). A Cell‐Free Platform Based on Nisin Biosynthesis for Discovering Novel Lanthipeptides and Guiding Their Overproduction *In Vivo* . Adv. Sci. 7, 2001616. 10.1002/advs.202001616 PMC7507342 32995136

[B27] McSweeney M. A. Styczynski M. P. (2021). Effective Use of Linear DNA in Cell-free Expression Systems. Front. Bioeng. Biotechnol. 9, 662. 10.3389/fbioe.2021.715328 PMC8329657 34354989

[B28] Mullin A. C. Slouka T. Oza J. P. (2022). “Simple Extract Preparation Methods for E. Coli-Based Cell-free Expression,” in *Cell-Free Gene Expression: Methods and Protocols* Methods in Molecular Biology. Editors Karim A. S. Jewett M. C. (New York, NY: Springer US), 51–64. 10.1007/978-1-0716-1998-8_2 34985736

[B29] Pardee K. Slomovic S. Nguyen P. Q. Lee J. W. Donghia N. Burrill D. (2016). Portable, On-Demand Biomolecular Manufacturing. Cell 167, 248–259. e12. 10.1016/j.cell.2016.09.013 27662092

[B30] Romantseva E. Strychalski E. A. (2020). CELL-FREE (Comparable Engineered Living Lysates for Research Education and Entrepreneurship) Workshop Report. Gaithersburg, MD: National Institute of Standards and Technology. 10.6028/NIST.SP.1500-13

[B31] Salis H. M. Mirsky E. A. Voigt C. A. (2009). Automated Design of Synthetic Ribosome Binding Sites to Control Protein Expression. Nat. Biotechnol. 27, 946–950. 10.1038/nbt.1568 19801975 PMC2782888

[B32] Shilling P. J. Mirzadeh K. Cumming A. J. Widesheim M. Köck Z. Daley D. O. (2020). Improved Designs for pET Expression Plasmids Increase Protein Production Yield in Escherichia coli. Commun. Biol. 3, 1–8. 10.1038/s42003-020-0939-8 32382055 PMC7205610

[B33] Shimizu Y. Inoue A. Tomari Y. Suzuki T. Yokogawa T. Nishikawa K. (2001). Cell-free Translation Reconstituted with Purified Components. Nat. Biotechnol. 19, 751–755. 10.1038/90802 11479568

[B34] Shimizu Y. Kanamori T. Ueda T. (2005). Protein Synthesis by Pure Translation Systems. Methods 36, 299–304. 10.1016/j.ymeth.2005.04.006 16076456

[B35] Si Y. Kretsch A. M. Daigh L. M. Burk M. J. Mitchell D. A. (2021). Cell-Free Biosynthesis to Evaluate Lasso Peptide Formation and Enzyme-Substrate Tolerance. J. Am. Chem. Soc. 143, 5917–5927. 10.1021/jacs.1c01452 33823110 PMC8062304

[B36] Silverman A. D. Karim A. S. Jewett M. C. (2020). Cell-free Gene Expression: an Expanded Repertoire of Applications. Nat. Rev. Genet. 21, 151–170. 10.1038/s41576-019-0186-3 31780816

[B37] Smith M. T. Berkheimer S. D. Werner C. J. Bundy B. C. (2014). Lyophilized Escherichia Coli-Based Cell-free Systems for Robust, High-Density, Long-Term Storage. BioTechniques 56, 186–193. 10.2144/000114158 24724844

[B38] Smith P. E. J. Slouka T. Dabbas M. Oza J. P. (2021). From Cells to Cell-Free Protein Synthesis Within 24 Hours Using Cell-Free Autoinduction Workflow. J. Vis. Exp. (173), e62866. 10.3791/62866 34369932

[B39] Swartz J. R. Jewett M. C. Woodrow K. A. (2004). “Cell-Free Protein Synthesis with Prokaryotic Combined Transcription-Translation,” in *Recombinant Gene Expression: Reviews and Protocols* Methods in Molecular Biology. Editors Balbás P. Lorence A. (Totowa, NJ: Humana Press), 169–182. 10.1385/1-59259-774-2:169 15269424

[B40] Williams L. C. Gregorio N. E. So B. Kao W. Y. Kiste A. L. Patel P. A. (2020). The Genetic Code Kit: An Open-Source Cell-free Platform for Biochemical and Biotechnology Education. Front. Bioeng. Biotechnol. 8, 941. 10.3389/fbioe.2020.00941 32974303 PMC7466673

[B41] Xu H. Yang C. Tian X. Chen Y. Liu W.-Q. Li J. (2022). Regulatory Part Engineering for High-Yield Protein Synthesis in an All-Streptomyces-Based Cell-free Expression System. ACS Synth. Biol. 11, 570–578. 10.1021/acssynbio.1c00587 35129330

[B42] Zhang L. Lin X. Wang T. Guo W. Lu Y. (2021). Development and Comparison of Cell-free Protein Synthesis Systems Derived from Typical Bacterial Chassis. Bioresour. Bioprocess. 8, 58. 10.1186/s40643-021-00413-2 34249606 PMC8258279

[B43] Zhang Y. Huang Q. Deng Z. Xu Y. Liu T. (2018). Enhancing the Efficiency of Cell-free Protein Synthesis System by Systematic Titration of Transcription and Translation Components. Biochem. Eng. J. 138, 47–53. 10.1016/j.bej.2018.07.001

