# Supplementary material for: Characterizing and Improving pET Vectors for Cell-free Expression
Source: Front Bioeng Biotechnol. 2022 Jun 23;10:895069. doi: 10.3389/fbioe.2022.895069 (PMC9259831; doi:10.3389/fbioe.2022.895069)
Supplement: Supplementary file 1 [file DataSheet1.docx]

Supplementary Materials

Characterizing and improving pET vectors for cell-free expression

Kara Jew^1^, Philip E.J. Smith^2^, Byungcheol So^2^, Jillian Kasman^2^, Javin P. Oza^2*^, Michael W. Black^1*^

^1^Biological Sciences Department, California Polytechnic State University, San Luis Obispo, CA, United States

^2^Chemistry & Biochemistry Department, California Polytechnic State University, San Luis Obispo, CA, United States

*** Correspondence:**
Javin P. Oza
joza@calpoly.edu

Michael W. Black
mblack@calpoly.edu

Contents:

Supplementary Figure S1

Supplemental Table 1

Supplementary Figure S1.


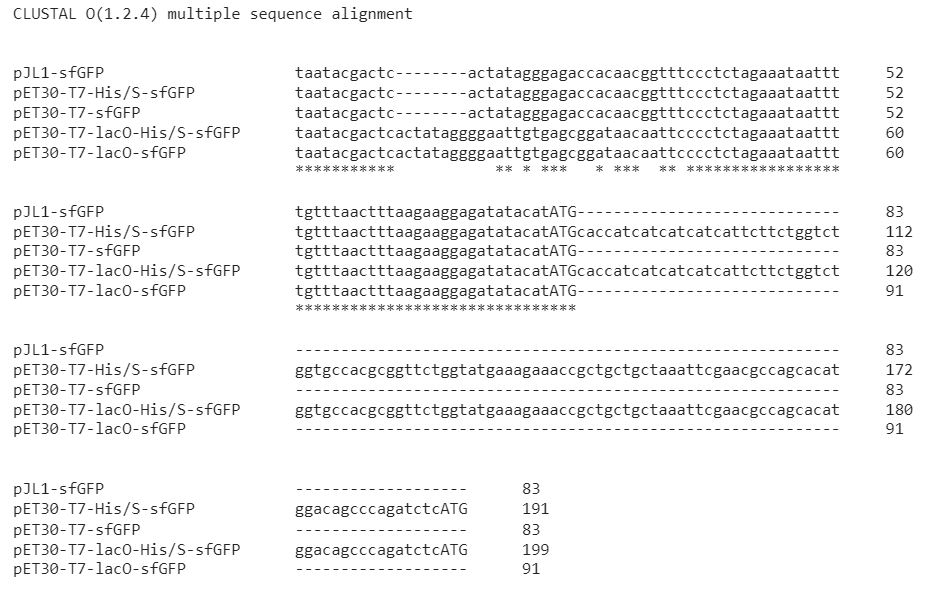


**Supplementary Figure S1. Sequence alignment of the vectors studied.** CLUSTAL Omega alignment of sequences from the five different plasmids evaluated in this study. The regions run from the 5’ end of the T7 promoter to the start codon of sfGFP. The sequence in this region is identical between the pJ1-sfGFP and pET30-T7-sfGFP, while the other pET30 versions include modifications representing the *lacO* sequence and the coding region for the N-terminal His and S tags.

Supplementary Table 1.

| Vector | Translation “Rate” | ΔG_total_ |
| --- | --- | --- |
| pJL1-sfGFP | 5,739 | -3.42 |
| pET30-T7-LacO-His/S-sfGFP | 21,843 | -6.39 |
| pET30-T7-LacO-sfGFP | 17,679 | -5.92 |
| pET30-T7-His/S-sfGFP | 9415 | -4.52 |
| pET30-T7-sfGFP | 5,739 | -3.42 |
